# Prevalence of suspected obesity hypoventilation syndrome in Hungarian Intensive Care Units during the COVID‐19 pandemic

**DOI:** 10.1111/crj.13667

**Published:** 2023-07-27

**Authors:** Szabolcs Baglyas, Luca Valkó, Dániel Donka, Gábor Fodor, Edit Hansági, István Méhész, János Gál, András Lorx

**Affiliations:** ^1^ Department of Anesthesiology and Intensive Care Semmelweis University Budapest Hungary; ^2^ Department of Anesthesiology, Intensive Care and Emergency Medicine Bajcsy‐Zsilinszky Hospital Budapest Hungary; ^3^ Central Department of Anesthesiology and Intensive Care Petz Aladár County Teaching Hospital Győr Hungary; ^4^ Department of Anesthesiology and Intensive Care Saint Barbara County Hospital Tatabánya Hungary; ^5^ Central Department of Anesthesiology and Intensive Care Uzsoki Street Hospital Budapest Hungary

**Keywords:** chronic respiratory failure, COVID‐19, ICU, obesity, OHS

## Abstract

**Introduction:**

The symptoms of obesity hypoventilation syndrome (OHS) may be present for years with concomitant progressive comorbidities, and the condition is frequently diagnosed late as a result of acute‐on‐chronic hypercapnic respiratory failure. Although some data exist on intensive care unit (ICU) prevalence, mortality and morbidity of OHS, little is known about the ICU mortality of these chronic respiratory failure patients during the COVID‐19 pandemic.

**Methods:**

We performed a cross‐sectional observational study in five Hungarian Intensive Care Units for 4 months during the COVID‐19 pandemic. All ICU patients were screened for OHS risk factors by treating physicians. Risk factors were defined as obesity (body mass index [BMI] ≥ 30 kg/m^2^) and at least one of the following: Epworth Sleepiness Score ≥ 6; symptoms of right heart failure; daytime or night‐time hypoxemia; presence of loud snoring; witnessed apnoea. We calculated prevalence, mortality and factors associated with unfavourable outcome.

**Results:**

A total of 904 ICU patients were screened for OHS risk factors. Overall 79 (8.74 ± 5.53%) patients were reported to have met the criteria for suspected OHS with a mortality rate of 40.5%; 69% (54 patients) of the cohort displayed at least 3 symptoms related to OHS before their acute illness. COVID‐19 infection was associated with higher mortality in OHS‐suspected patients, independently of actual BMI.

**Conclusion:**

Despite the increased risk of obese patients, suspected OHS did not show higher prevalence than expected during the COVID‐19 pandemic in critically ill patients. COVID‐19 infection however was a risk for mortality in these patients, independent of actual BMI.

## Graphical abstract

We conducted an observational study in Hungarian Intensive Care Units during the COVID‐19 pandemic to uncover OHS prevalence and related outcome. Out of 904 patients screened, 79 (8.74 ± 5.53%) patients met the criteria for suspected obesity hypoventilation syndrome (OHS). This is a relatively low prevalence given the reported increased rates of obesity among critically ill COVID‐19 patients. COVID‐19 infection was associated with higher mortality in our cohort of OHS‐suspected patients, independently of actual BMI.

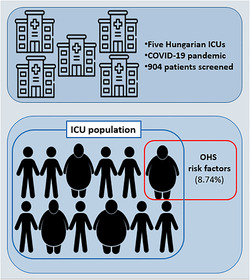

## 1 INTRODUCTION

The growing rates of obesity worldwide pose several clinical challenges, including the emergence of obesity hypoventilation syndrome (OHS) as a leading cause of chronic respiratory impairment.^1^, ^2^ OHS is defined as the combination of sleep‐related hypoventilation, resting daytime hypercapnia (arterial carbon dioxide [P_a_CO_2_] ≥ 45 Hgmm) and a body mass index (BMI) of ≥30 kg˙m^−2^ in the absence of alternative cause for alveolar hypoventilation.^3^ OHS is also associated with significant morbidity, leading to increased rates of chronic heart failure, pulmonary hypertension and metabolic syndrome.^4^ Although symptoms indicative of the condition and its comorbidities may be present for years, OHS is frequently diagnosed late in its course as acute‐on‐chronic hypercapnic respiratory failure.^5^, ^6^, ^7^, ^8^ As such, intensive care units (ICUs) are an important healthcare setting for patients with OHS.

Data are limited regarding the prevalence of OHS in the ICU and its effect on mortality in different settings. While the overall prevalence of obesity is 20–30% in ICUs, a previous retrospective monocentric study found that 8% of ICU admissions met the criteria of OHS and extreme obesity (BMI > 40 kg˙m^−2^).^9^ Both the obese and OHS patients may benefit from the obesity paradox, which results in similar or even decreased mortality rates compared with non‐obese patients.^5^, ^10^, ^11^, ^12^ This protective effect was seemingly diminished by COVID‐19. During the pandemic, the obese made up the majority of critically ill patients (up to 48%) and were more likely to require invasive mechanical ventilation and die (with a mortality rate of 28–35%).^13^, ^14^, ^15^, ^16^ Despite extensive data on outcomes for the obese in COVID‐19, little is known about the ICU prevalence of OHS and the mortality of this cohort during the COVID‐19 pandemic.

The current study aimed to assess suspected OHS prevalence and associated outcomes among critically ill patients during the COVID‐19 pandemic. We conducted a multicenter cross‐sectional investigation in general ICUs. Primary outcomes were the prevalence and mortality of suspected OHS in critically ill patients. Secondary outcomes were relative risk of suspected OHS prevalence and mortality due to COVID‐positive status, pneumonia or need for invasive ventilation (IV).

## 2 MATERIALS AND METHODS

### 2.1 Design of the study

Eleven high case number (minimal bed number of 12), mixed case patient population ICUs participating in COVID‐19 management were invited to take part in the study. Out of the 11, 5 ICU sites took part in the study. Investigating physicians were asked to screen all critical care patients for risk factors of OHS during the study period between the 1st of October and the 30th of November, 2020 and again between the 1st of October and 30th of November, 2021. Overall patient number, COVID‐19 prevalence and mortality were recorded for all participating units during the study period. Patients qualifying as suspected OHS were included in the study. Patients were excluded if they developed OHS‐related symptoms due to acute illness. We also excluded patients aged under 18 years. Written informed consent was provided by all participating patients or their next of kin. Data were collected anonymously. The study protocol was approved by the regional ethical committee of Semmelweis University (SE RKEB 52/2020.).

### 2.2 Screening

Suspected OHS was defined as a BMI of ≥30 kg/m^2^ and the presence of at least one OHS‐related risk factor before the current acute illness (Table 1).^2^

**TABLE 1:** OHS‐related risk factors.^2^

Daytime sleepiness
Symptoms of right heart failure
Daytime hypoxemia on room air (Sat 92–95%)
Nighttime hypoxemia on room air (Sat < 90%)
Loud snoring
Witnessed apnoea

Abbreviation: OHS, obesity hypoventilation syndrome.

Epworth Sleepiness Score was calculated to evaluate daytime sleepiness; a score of ≥6 was considered abnormal.^17^ Symptoms of right heart failure were considered according to European Society of Cardiology guidelines for the diagnosis and treatment of acute and chronic heart failure.^18^ Daytime and nighttime hypoxemia was based on previous outpatient or in‐hospital medical records. The presence of loud snoring and witnessed apnoea was based on history collected from next of kin. In case of missing data, the risk factor was assumed to be absent.

### 2.3 Data collection

Data collected included anthropometry data (sex, age, BMI), past medical history (comorbidities, previous hospitalization), COVID‐19 status, ICU length of stay (LOS), length of invasive ventilation (LOV), ICU mortality, ICU readmission rate and abnormal arterial blood gas values indicative of hypoventilation (hypercapnia, elevated bicarbonate).

Anthropometry data were collected by critical care physicians based on the medical records of the patients and information given by the patients or relatives. BMI was calculated on admission.

Information about past medical history, comorbidities such as diabetes, congestive heart disease, pulmonary hypertension and ischaemic heart disease, was based on information provided by healthcare professionals, information in previous medical reports and information based on findings during critical care treatment. Chronic kidney disease nomenclature was used according to Kidney Disease Improving Global Outcomes 2012 Clinical Practice Guidelines for the Evaluation and Management of Chronic Kidney Disease.^19^ Hospital admission and ICU admission data in the last 12 months were based on the electronic medical record of the patient. COVID‐19 status was based on a polymerase chain reaction (PCR) test (sputum, tracheal aspirate or nasopharyngeal swab) performed at any time during the course of the acute illness resulting in the study hospital admission. The total number of days spent in the ICU defined the ICU LOS. The total number of days spent with invasive respiratory support defined LOV. Hypercapnia (p_a_CO_2_ ≥ 45 mmHg) and increased bicarbonate (HCO_3_^−^) levels (≥27 mmol/L) on arterial blood gas measured at the time of discharge from ICU defined alveolar hypoventilation.^11^

### 2.4 Statistical analysis

The results are expressed as median (±standard deviation) for continuous variables and as frequency (percentage) for categorical variables. Different patient groups (OHS suspected vs. OHS not suspected, COVID‐19 positive vs. negative patients, IV required vs. not required, survivors vs. non‐survivors, different BMI and age groups) were compared with 2 × 2 or 3 × 2 Pearson's Chi‐square. *P* < 0.05 value was considered significant. All statistical analyses were performed with SPSS (IBM Corp., Armonk, NY).

## 3 RESULTS

The five participating ICUs treated a total of 904 patients during the study period. Overall mortality reported by the units was 35.40%. Overall COVID‐19 prevalence of the ICUs was 58.96% during the study period.

**FIGURE 1:**
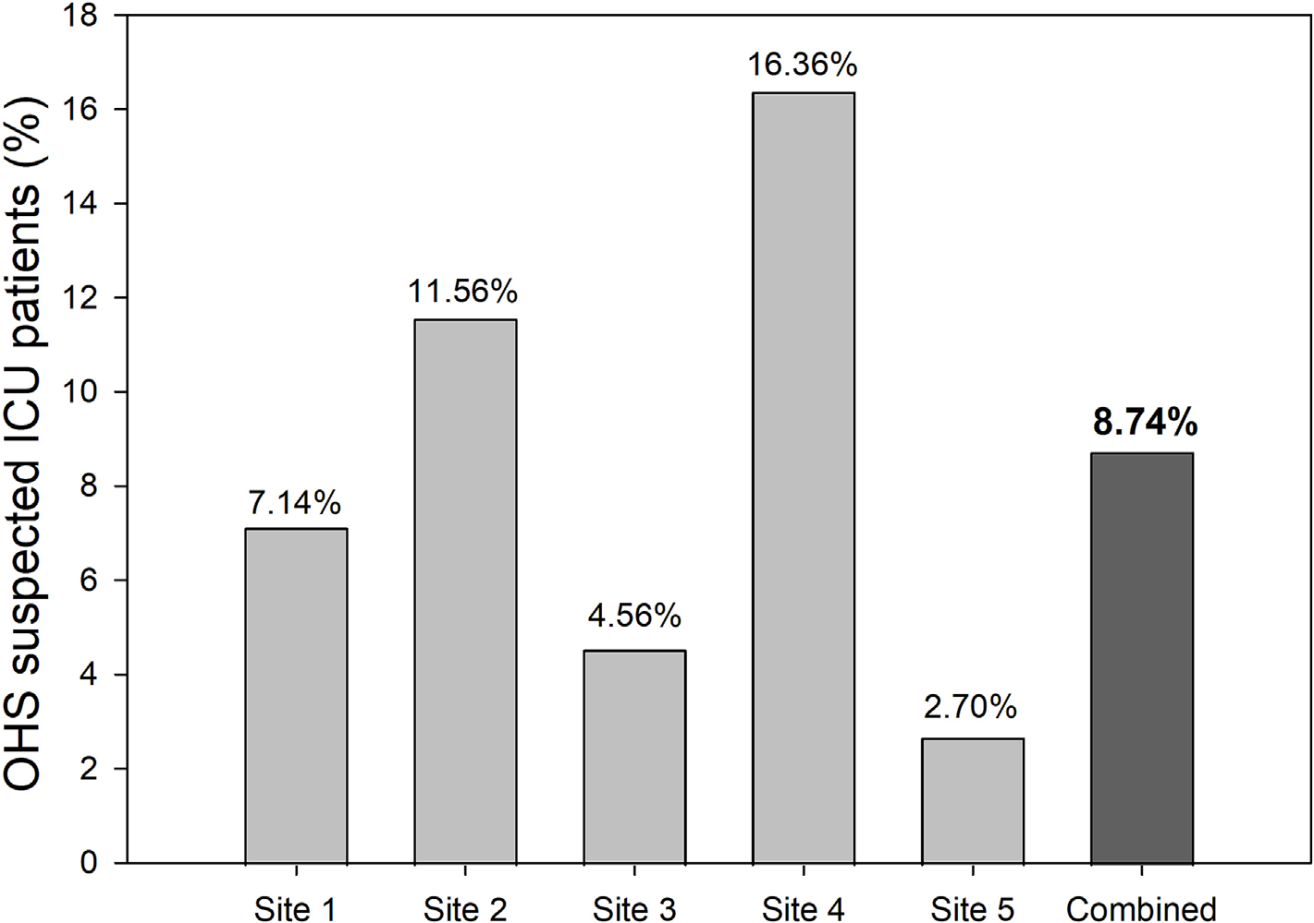
The overall prevalence of patients with suspected obesity hypoventilation syndrome (OHS) in intensive care unit (ICU).

Out of the 904 ICU patients screened, 79 patients (8.74 ± 5.53%) were reported to have risk factors for OHS. Prevalence of suspected OHS varied by site, and a significant difference was observed between ICU sites (*p* < 0.001) (see Figure 1).

**FIGURE 2:**
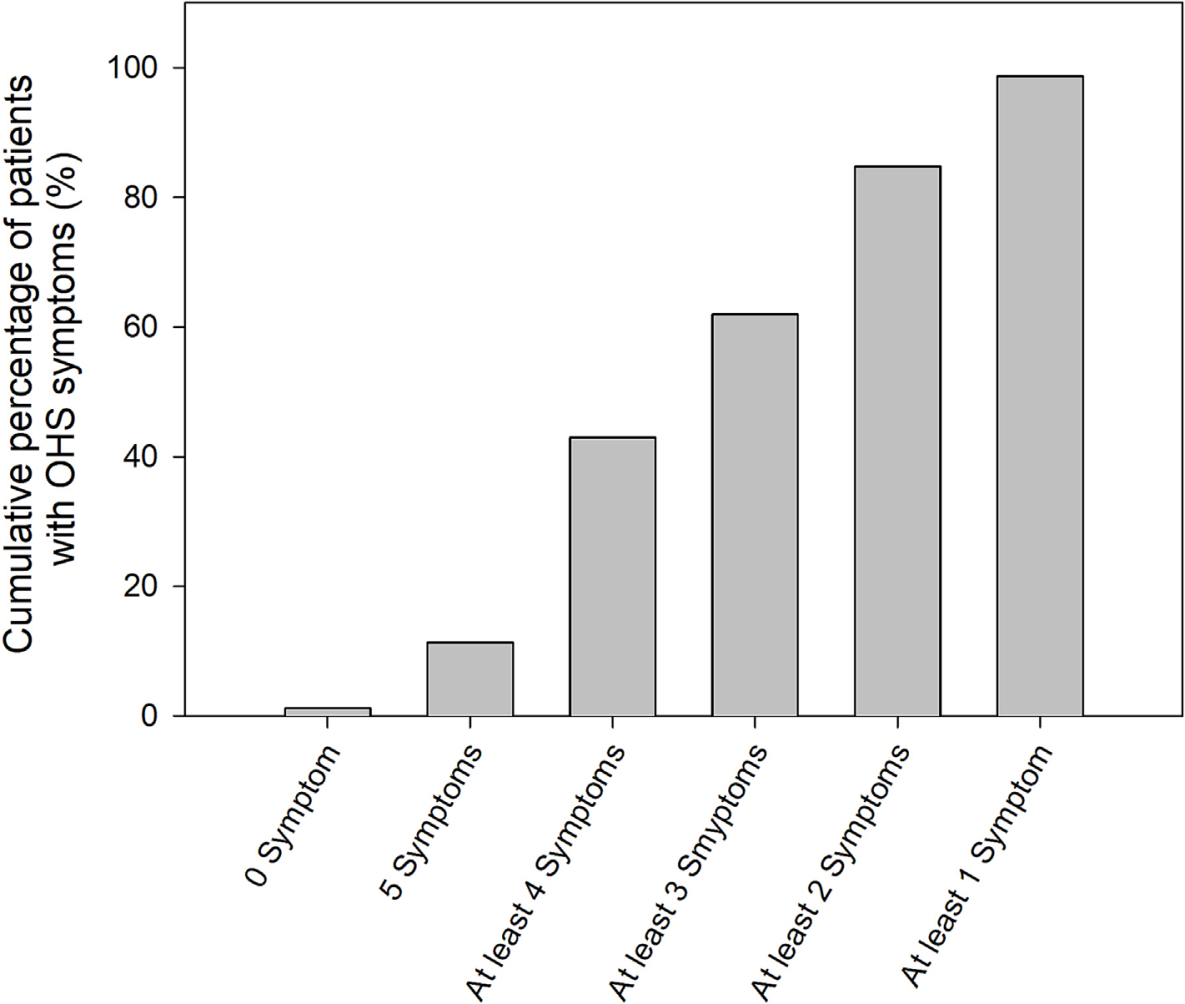
Cumulative percentage of obesity hypoventilation syndrome (OHS) suspected intensive care unit (ICU) patients with OHS‐related symptoms. OHS‐related symptoms: Epworth Sleepiness Score ≥ 6; pitting oedema; nocturia; loud snoring; witnessed apnoea.

Daytime sleepiness scores were reported for 55 out of 79 patients and were abnormal for 45 patients (81.8%) (Table 2). Snoring, nocturia and pitting oedema were the most frequent symptoms among the reported individuals (86.1%, 69.6% and 65.8%, respectively). Seventeen patients (21.5%) had witnessed apnoea by relatives; 69% of the cohort displayed at least 3 symptoms related to OHS (Figure 2).

**TABLE 2:** The clinical characteristics of OHS‐suspected patients (mean, SD/*N*, %).

Anthropometry	
Total (*N*, %)	79 (100%)
Male (*N*, %)	57 (72.20%)
Female (*N*, %)	22 (27.80%)
Age (years) (Mean, SD)	62.94 (±11.91)
BMI (kg/m^2^) (Mean, SD)	37.63 (±6.40)
Obesity WHO Class I (*N*, %)^20^	33 (41.8%)
Obesity WHO Class II (*N*, %)	24 (30.4%)
Obesity WHO Class III (*N*, %)	22 (27.8%)
ICU LOS (days) (Mean, SD)	11,52 (±9.27)
Invasive ventilation required (*N*, %)	60 (75.9%)
LOV (days) (Mean, SD)	8.67 (±9.51)
Mortality (*N*, %)	32 (40.50%)
COVID positive (*N*, %)	38 (48.10%)
OHS‐related symptoms^11^ (*N* = 59)	
Epworth Sleepiness Score > 5 (*N*, %)	49 (83.05%)
Pitting oedema (*N*, %)	52 (65.8%)
Nocturia (*N*, %)	55 (69.6%)
Loud snoring (*N*, %)	68 (86.1%)
Witnessed apnoea (*N*, %)	17 (21.5%)
Comorbidities^2^	
Congestive heart failure (*N*, %)	27 (34.2%)
Pulmonary hypertension (*N*, %)	10 (12.7%)
Ischaemic heart disease (*N*, %)	19 (24.1%)
Diabetes mellitus (*N*, %)	33 (41.8%)
Chronic renal failure (*N*, %)	7 (8.9%)
No comorbidities (*N*, %)	27 (34.2%)
Hospital admission in the last 12 months (*N*, %)	23 (29.1%)
Diagnosis at admission	
Pneumonia (*N*, %)	46 (58.2%)
Congestive heart failure (*N*, %)	12 (15.2%)
Acute exacerbation of COPD (*N*, %)	6 (7.6%)
Acute kidney failure (*N*, %)	3 (3.8%)
Pulmonary embolism (*N*, %)	3 (3.8%)
St. p. CPR (*N*, %)	3 (3.8%)
Other nonsurgical (*N*, %)	4 (5.1%)
Surgical/trauma (*N*, %)	10 (12.7%)

Abbreviations: BMI, body mass index; COPD, chronic obstructive pulmonary disease; CPR, cardiopulmonary resuscitation; ICU, intensive care unit; LOS, length of stay; LOV, length of invasive ventilation; OHS, obesity hypoventilation syndrome.

Characteristics of the study patients are listed in Table 2. More than two‐third of suspected OHS patients (72.2%) were male, and the mean age was 62.94 (±11.91) years. The two most frequent indications for ICU admission were pneumonia (46 patients, 58.2%) and congestive heart failure (12 patients, 15.2%). Almost half of the patients (48.1%) had confirmed SARS‐CoV‐2 infection. On the day of discharge, 14 (29.87%) survivors had elevated HCO_3_^−^, and 12 (25.53%) had elevated p_a_CO_2_ levels despite normal pH, indicating potential hypoventilation. Ten (21.27%) patients had confirmed overnight hypoventilation and elevated p_a_CO_2_ levels during spontaneous breathing.

LOS was 11.52 (±9.27), and LOV was 8.67 (±9.51). Mortality rate for suspected OHS patients was 40.5%. There was no reported readmission of suspected OHS patients during the study period.

As Table 3 shows, the mortality rate did not differ in OHS‐suspected patients compared to the ICU patients without OHS risk factors. The mortality rate was similar in both study periods (37.8% vs. 40%; *p* > 0.05). In the suspected OHS group, patients with COVID‐19 infection and pneumonia had increased mortality (OR 17.67; 95% CI 5.49–56.88; *p* < 0.001 and OR 11.28; 95% CI 3.39–37.50; *p* < 0.001, respectively) and a higher rate of invasive mechanical ventilation (OR 7.47; 95% CI 1.97–28.39; *p* = 0.001 and OR 4.33; 95% CI 1.43–13.1; *p* = 0.007, respectively) compared with those without these factors (Table 4).

**TABLE 3:** Combined mortality rate in the ICU sites and among the OHS suspected patients.

ICU cohort	Overall (*N* = 904)	OHS suspected (*N* = 79)	OHS not suspected (*N* = 825)	OR (CI 95%)	*P* *x* ^2^ test
*Mortality*	320 (35.4%)	32 (40.5%)	288 (34.9%)	1.270 (0.792–2.034)	0.988

Abbreviations: AE‐COPD, acute exacerbation of chronic obstructive pulmonary disease; ICU, intensive care unit; OHS, obesity hypoventilation syndrome.

**TABLE 4:** The impact of anthropometric data, admitting diagnosis and past medical history on outcome.

	All suspected OHS patients *N* = 79	ICU survivors *N* = 47	Non‐survivors *N* = 32	OR (CI 95%)	*P* *x* ^2^ test
Anthropometry					
Sex (male)	57 (72.2%)	33 (70.2%)	24 (75%)	0.79 (0.29–2.17)	0.64
Age > 65 years	35 (44.3%)	20 (42.6%)	15 (46.9%)	1.91 (0.48–2.9)	0.70
ICU diagnosis					
Pneumonia	46 (58.2%)	18 (39.1%)	28 (87.5%)	11.28 (3.39–37.50)	**<0.001**
Congestive heart failure	12 (15.2%)	11 (23.4%)	1 (3.1%)	0,11 (0,01‐0,86)	**0.01**
AE‐COPD	6 (7.6%)	6 (12.8%)	0 (0%)	0.87 (0.78–0.97)	**0.03**
Acute kidney failure	3 (3.8%)	3 (6.4%)	0 (0%)	0.94 (0.87–1.01)	0.15
Pulmonary embolism	3 (3.8%)	3 (6.4%)	0 (0%)	0.94 (0.87–1.01)	0.15
Post cardiac arrest	3 (3.8%)	1 (2.1%)	2 (6.3%)	3.07 (0.27–35.33)	0.35
Other non‐surgical	4 (8.5%)	4 (8.5%)	0 (0%)	0.915 (0.84–0.99)	0.09
Surgical/trauma	10 (12.7%)	9 (19.1%)	1 (3.1%)	0.14 (0.16–1.13)	**0.04**
COVID‐19	38 (48.1%)	11 (23.4%)	27 (84.4%)	17.67 (5.49–56.88)	**<0.001**
Medical history					
Chronic heart failure	27 (34.2%)	18 (38.3%)	9 (28.1%)	0.63 (0.239–1.66)	0.35
Pulmonary hypertension	10 (12.7%)	5 (10.6%)	5 (15.6%)	1.56 (0.41–5.89)	0.51
Ischaemic heart disease	19 (24.1%)	8 (17.0%)	11 (34.4%)	2.55 (0.89–7.33)	0.08
Diabetes mellitus	33 (41.8%)	16 (34.0%)	17 (53.1%)	2.20 (0.88–5.51)	0.09
Chronic kidney disease	7 (8.9%)	4 (8.5%)	3 (8.9%)	1.11 (0.23–5.34)	0.90

Abbreviations: ICU, intensive care unit; OHS, obesity hypoventilation syndrome.

**FIGURE 3:**
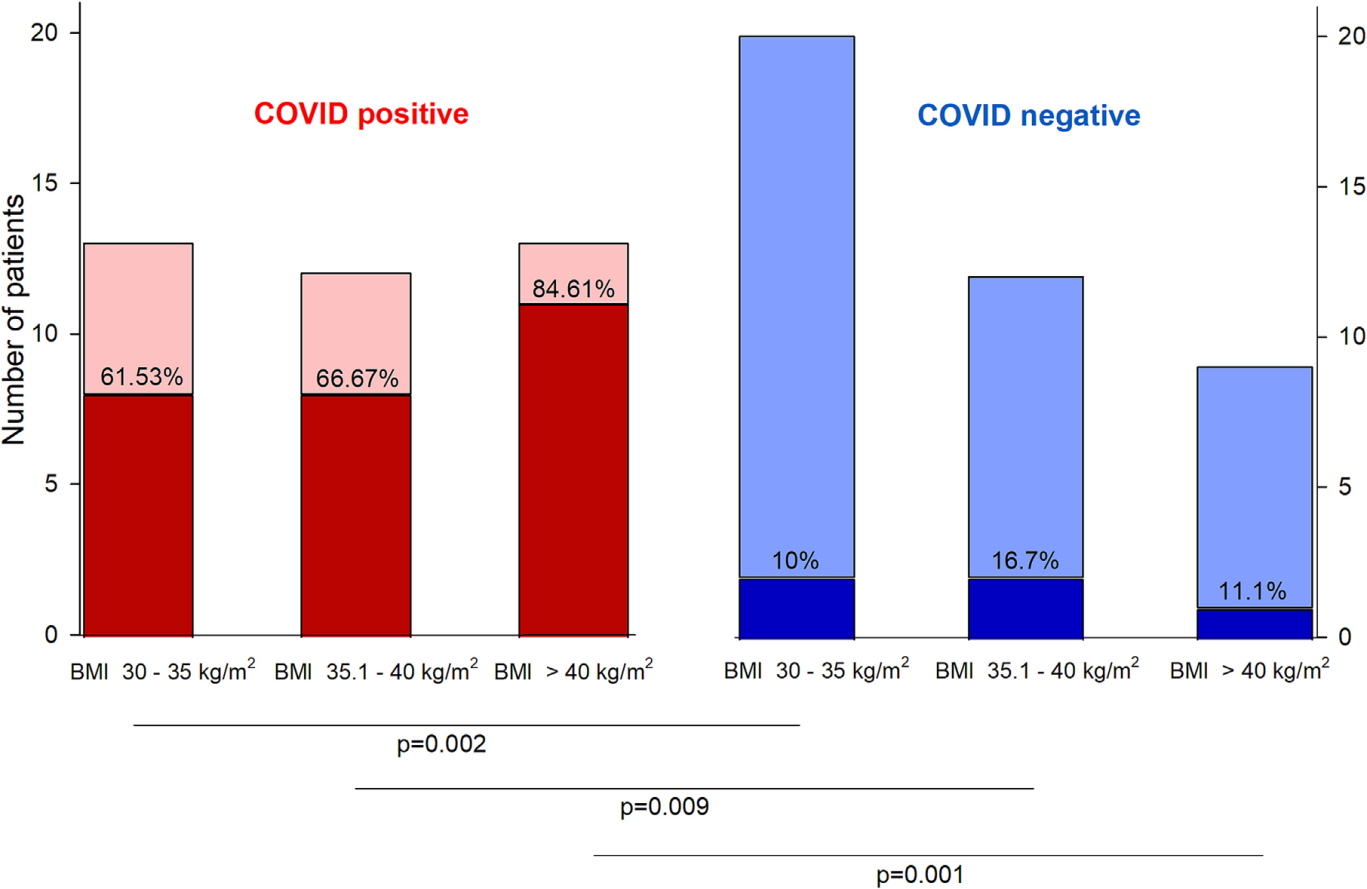
Impact of COVID‐19 on the mortality of obesity hypoventilation syndrome (OHS) suspected intensive care unit (ICU) patients. Red bars indicate the number of patients with COVID‐19 infection in each body mass index (BMI) category, and blue bars correspond to COVID‐19 negative patients. Dark shading represents the mortality rate in each category.

COVID‐19 infection increased mortality in each BMI category [BMI 30–35 kg.m^−2^ OR 14.4; 95% CI 2.29–90.60; BMI 35.1–40 kg.m^−2^ OR 11.0 95% CI 1.60–75.50; BMI > 40 kg.m^−2^ OR 38.5 95% CI 2.91–508.46]. However, in the COVID‐19‐positive cohort, there was no difference in mortality rate based on BMI categories (*p* = 0.374) (Figure 3).

## 4 DISCUSSION

Our multicentre, cross‐sectional observational study aimed to assess the prevalence and mortality of suspected OHS in ICUs during the COVID‐19 pandemic. The mean prevalence of suspected OHS was 8.74% in our examined sites, with patients showing frequent persisting symptoms, and potential signs of chronic hypoventilation at discharge. Mortality of patients with suspected OHS was similar to patients without OHS risk factors but increased significantly if COVID‐19 infection was present.

The overall prevalence of OHS is 0.15–0.3% in the general population.^2^ This data are based on the United States (US) population and is highly dependent on the prevalence of obesity. Obesity rates are documented to be lower in Europe but are closer to US rates in Hungary (US: 27–32%; Europe: 23.3%; Hungary: 26.4%).^1^, ^21^ A previously cited study from Marik et al. observed that 8% of the ICU population met the criteria of OHS in the United States; however, they used BMI ≥ 40 kg/m^2^ as inclusion criteria.^9^ COVID‐19 greatly increased the prevalence of obesity in the ICU population, suggesting a subsequent increase in OHS prevalence as well during this period.^22^ Despite this, we found an overall 8.74% prevalence of suspected OHS among ICU patients during the COVID‐19 pandemic, with about 50% of our study patients presenting as COVID‐19 negative. This suggests that COVID‐19 infection did not result in increased ICU admission of OHS‐suspected patients.

As previous studies have shown, patients with obesity are admitted to the ICU frequently, and increased BMI categories are associated with comorbidities such as diabetes mellitus, cardiac failure and respiratory failure.^23^ During the COVID‐19 pandemic, the prevalence of obesity in ICUs reached almost 50%; moreover, increased mortality rate and frequent need for invasive mechanical ventilation were observed among obese and severely obese patients.^15^, ^22^ In our study, both pneumonia and COVID‐19 infection were risk factors for mortality and the need for invasive mechanical ventilation. However, the actual mortality rate was independent of BMI categories. These findings suggest that the presence of the risk factors for OHS and chronic respiratory failure may carry an increased risk in COVID‐19 independently of the actual body weight.

Among critically ill patients, obesity may be associated with greater survival.^23^ This paradoxical protective effect was apparent in OHS‐suspected patients as well^13^ but was negatively affected by COVID‐19.^22^ In our study, OHS‐suspected patients displayed higher mortality rates than was expected by the obesity paradox. This was mainly driven by COVID‐19 infections, as suspected OHS patients without COVID‐19 had similar mortality rates to previously reported historical data (10–16.7%).^12^, ^23^ We also found that COVID‐19‐positive status increased mortality independent of actual BMI. These data suggest that the presence of suspected OHS is a risk factor for mortality in COVID‐19 positive patients independent of actual body weight. This is a strong indication that obese patients with risk factors of chronic respiratory failure may be more fragile during the pandemic.

As previous studies highlighted, OHS patients are predominantly diagnosed late, during an episode of acute hypercapnic respiratory failure.^11^ It is also known that some of these patients, even in this stage, are falsely diagnosed with chronic obstructive pulmonary disease (COPD) rather than OHS.^9^ Our study highlighted that more than 60% of OHS‐suspected patients carry at least three symptoms connected to OHS besides obesity. It is also noteworthy that more than 60% of patients had at least one documented comorbidity related to OHS, meaning OHS could have been suspected and diagnosed before their current hospitalization.^2^ There is an urgent need to draw attention to this population of patients who might benefit from advanced screening for OHS.

Additionally, even before the pandemic, the mortality of ICU survivors with subsequently untreated OHS was 24–46% in the years following ICU discharge.^2^, ^11^ According to the American Thoracic Society guideline, OHS patients with persisting hypercapnia should receive non‐invasive ventilation (NIV) after critical care treatment of a hypercapnic respiratory failure episode until further evaluation in a sleep laboratory.^4^ It has been shown that long‐term NIV treatment initiated after acute hospitalization can improve the quality of life significantly in this patient group.^24^ Almost one‐third of survivors in our study had elevated p_a_CO_2_ and/or HCO_3_^−^ levels at the time of discharge from the ICU, potentially qualifying them for long‐term respiratory support,^25^, ^26^ which can improve survival and quality of life.^24^ This reinforces that ICUs are important sites for flagging patients with suspected OHS.

In summary, we found that the overall ICU prevalence of suspected OHS during the COVID‐19 pandemic was similar to historical data, despite the higher prevalence of the obese in critically ill patients. Several reasons may be suspected behind this apparent ‘protective’ effect, most obviously that multimorbid patients were more likely to take precautionary measures and avoid infection during the pandemic. Mortality of COVID‐19‐positive OHS suspected patients was significantly higher than mortality of COVID‐19‐negative patients, independent of BMI categories, highlighting the fragility of this population during the pandemic.

Despite these important findings, our study has some limitations. The relatively short observational period might have influenced our results, as seasonal fluctuation of patients with respiratory insufficiency in ICUs is well known.^27^ The markedly different prevalence of suspected OHS at the different study sites might indicate different clinical practices during the pandemic, but our study did not focus on uncovering reasons for this occurrence. Additionally, the study was executed during the second and fourth wave of the COVID‐19 pandemic in Hungary with different dominant strains, which might have influenced the prevalence of obesity, and mortality rates observed in our study.

## 5 CONCLUSION

In conclusion, we found that the prevalence of suspected OHS in ICUs was similar during the COVID‐19 pandemic compared with historical data, despite the well‐known increased prevalence of obesity in critically ill patients during this time. The mortality rate for the COVID‐19‐positive suspected OHS population was high (40.5%) and independent of BMI. This suggests that suspected OHS was protective from hospitalization with critical illness during the pandemic but was a risk factor for death once COVID‐19 infection occurred. Further studies are needed to confirm and explain the potential reasons for these observations.

## AUTHOR CONTRIBUTIONS

Sz. Baglyas, L. Valkó and A. Lorx designed the study. Sz. Baglyas, L. Valkó, D. Donka, G. Fodor, E. Hansági, I. Méhész and J. Gál recruited patients and collected data. Sz. Baglyas, L. Valkó and A. Lorx analysed and interpreted the data. Sz. Baglyas, L. Valkó, A. Lorx and J. Gál drafted the manuscript. All authors have seen and approved the submitted manuscript.

## CONFLICT OF INTEREST STATEMENT

The authors declare that they have no relevant or material financial interests that relate to the research described in this paper.

## ETHICS APPROVAL AND CONSENT TO PARTICIPATE

The study was approved by the research ethics board of Semmelweis University (SE RKEB 52/2020). Participation was voluntary, and data were collected anonymously. All procedures performed involving human participants were in accordance with the ethical standards of the institutional and/or national research committee and with the 1964 Helsinki Declaration and its later amendments or comparable ethical standards.

## Data Availability

The datasets generated and analysed during the current validation study are available from the corresponding author on reasonable request.
